# Walking on an unpredictable irregular surface changes lower limb biomechanics and subjective perception compared to walking on a regular surface

**DOI:** 10.1186/1757-1146-7-S1-A81

**Published:** 2014-04-08

**Authors:** Thorsten Sterzing, Charlotte Apps, Rui Ding, Jason Tak-Man Cheung

**Affiliations:** 1 Sports Science Research Center, Li Ning (China) Sports Goods Co Ltd, Beijing 101111, China; 2 School of Sport and Exercise Sciences, Liverpool John Moores University, Liverpool, L3 3AF, UK

## Background

Irregular surface conditions, for instance, are present during trail walking. Modified treadmills can be used to produce such surface conditions in a laboratory environment [1]. Walking on an irregular surface showed increased gait variability [2], which is regarded as a beneficial training stimulus [3]. Thus, this study examined the effects of an unpredictable irregular surface (UIS) on lower limb biomechanics, locomotion variability, and subjective perception during treadmill walking.

## Methods

Seventeen young, male, active participants walked at 5 km/h on a treadmill with predictable regular surface (PRS) and with UIS. The UIS was created by randomly attaching EVA dome shaped inserts (ط: 140 mm) of different height (10 mm and 15 mm) and hardness (40 and 70 Asker C) to the treadmill. In-shoe plantar pressures (200 Hz, Pedar X System, Novel, Germany), lower limb kinematics (200 Hz, Vicon Peak, United Kingdom), and EMG signals of five lower limb muscles (3000 Hz, Telemyo 2400 G2, Noraxon, USA) were recorded. Eight perception items were assessed subjectively (9-point Likert Scale). Biomechanical parameter mean magnitudes and mean standard deviations, as variability measure, of 16 steps were calculated. Variables were compared between surfaces by Wilcoxon signed rank tests (p<.05).

## Results

Step length increased, while step frequency decreased on UIS (p<.05). In-shoe pressure relative load magnitudes were consistent between conditions for five out of six masks, with only the medial midfoot loaded higher on UIS (p<.05). Relative load variability increased on UIS for all masks (p<.05). Small but significant kinematic differences at touchdown were found, with markedly greater variability on UIS: Reduced shoe-surface angle and ankle dorsiflexion, increased knee and hip flexion. The ankle joint showed decreased inversion at touchdown and increased maximum eversion on UIS, alongside higher variability (Table 1). Whereas muscle activity magnitude was similar for tibialis anterior and gastrocnemius medialis on both surfaces, it was increased for peroneus longus on UIS. In contrast, muscle activity variability was increased for tibialis anterior and gastrocnemius medialis on UIS, whereas it was similar for peroneus longus (Table 1). Subjectively, walking on UIS was more challenging (p<.05).

**Table 1:** Magnitude (Mag) and variability (Var) of kinematic and EMG parameters, significant surface comparisons (PRS vs. UIS) indicated in bold.

	Frontal plane ankle angle [deg]				Normalized muscle activity during stance [%]					
	
	Inversion touchdown		Eversion maximum		Tibialis Anterior		Gastrocnemius Med		Peroneus Longus	
	Mag	Var	Mag	Var	Mag	Var	Mag	Var	Mag	Var

**PRS**	-2.8	1.5	7.7	0.9	19.1	2.6	31.3	4.9	38.8	9.7

**UIS**	-1.5	1.9	9.3	3.4	19.6	3.4	31.9	5.5	48.5	11.3

**p-value**	**.006**	**.002**	**.001**	**001**	.435	**.013**	.831	**.049**	**.006**	.163

## Conclusion

On UIS, muscle specific motor control strategies were applied. Frontal plane stabilization effort of the ankle joint was consistently increased throughout all ground contacts. Sagittal ankle joint mobilization and/or stabilization depended on specific perturbation effects of single ground contacts. Walking on UIS induced a more variable gait, thus stimulating enhancement of motor control patterns, resembling a positive training mechanism [4].
